# Comparison of the Physical and Mechanical Properties of Resin Matrix with Two Photoinitiator Systems in Dental Adhesives

**DOI:** 10.3390/polym8070250

**Published:** 2016-07-01

**Authors:** Mijoo Kim, Byoung-In Suh, Daehwan Shin, Kwang-Mahn Kim

**Affiliations:** 1 Department & Research Institute of Dental Biomaterials & Bioengineering, College of Dentistry, Yonsei University, 50-1 Yonsei-ro, Seodaemun-gu, Seoul 03722, Korea; mijookim@yuhs.ac; 2 Bisco, Inc., 1100 W, Irving Park Road, Schaumburg, IL 60193, USA; bisuh@bisco.com (B.-I.S); dhshin@bisco.com (D.S.)

**Keywords:** dental adhesives, degree of conversion, iodonium salts, microtensile strength, dynamic microhardness, ternary photoinitiator system

## Abstract

This study evaluated the physical and mechanical properties of resin matrices in dental adhesives with two photoinitiator systems. Resin matrix specimens were made with five different kinds of photoinitiators. Neat resin consisted of 60% 2,2-bis[4-2(2-hydroxy-3-methacryloxypropoxy)phenyl]propane (Bis-GMA) and 40% hydroxyethyl methacrylate (HEMA) by weight, along with camphorquinone (CQ, 1 mol %) and additional components (1 mol % each) as follows: Group 1, 2-(dimethylamino)ethyl methacrylate (DMAEMA); Group 2, ethyl-4-(dimethylamino) benzoate (EDMAB); Group 3, diphenyliodonium hexafluorphosphate (DPIHFP); Group 4, DMAEMA+DPIHFP; Group 5, EDMAB+DPIHFP. The degree of conversion (DC), flexural strength, flexural modulus, microhardness, and ultimate tensile strength were tested. The contribution of each photoinitiator to the DC in a selected group was analyzed with contour plots. One-way ANOVA and Tukey tests (*p* < 0.05) were used for statistical analyses. The DC of Groups 2, 4, and 5 was similar. The flexural strength was similar in all groups, but flexural modulus was significantly different. Group 3 had the lowest values for all physical and mechanical properties. Among all methods, the microhardness test revealed the greatest degree of difference among the five specimens. CQ, EDMAB, and DPIHFP were the most effective photoinitiators and CQ was the most influential factor for the DC rate.

## 1. Introduction

Since dental resin technology was first introduced over 50 years ago, its use has recently increased due to patients’ demands for esthetic restorations and clinicians’ needs for easy and direct application. It enables clinicians to follow a predictable, conservative, and reliable chairside protocol for enhancing patient smiles and restoring worn and decayed tooth structure. The ability to be minimally invasive and conserve tooth structure is another benefit in the use of dental resin. Therefore, products with resin components can be used on a daily basis to restore caries, close spaces, lengthen teeth, cover dark or discolored teeth, and fix fractured teeth.

The longevity of dental restorative composites is a critical concern for the long-lasting retention of restorations and depends on the stability of the hybrid layer formed between the composite resin and the dentin/enamel structure. Therefore, adhesive resins with an effective polymerization system are needed for the best retention of the hybrid layer. The adhesive or bonding resin promotes bonding between enamel or dentin and the resin composite restorative material while simultaneously producing the stable hybrid layer that increases longevity. However, these resins contain relatively less filler than packable or flowable restorative resins used for low-viscosity applications. As a result, they can be more easily affected by the physical and mechanical properties of the resin matrix [1,2]. Manufacturers add nanofillers to the matrix mixture to reduce shrinkage during polymerization and water sorption and to improve its mechanical properties. However, the filler content of adhesives is approximately 10 wt % [3], which is much less than those of restorative composites which are approximately 60–80 wt % [4]. Therefore, investigations of the resin matrix are necessary for producing stable and solid dental adhesives.

Ideally, all monomer molecules in the resin matrix are converted to the polymer during the polymerization reaction. However, dimethacrylate monomers exhibit residual (C=C) bonds in the final product with the degree of conversion (DC) ranging from 55% to 75% under conventional irradiation conditions [5,6]. These residual bonds result in a poor mechanical strength which cause fractures in the dental filling resin or leakage between the tooth and filling resin above the adhesives within the cavity. In addition, the release of toxic monomers to dentinal tubules and detached surfaces induces toxicity to the tooth and oral environment and accelerates the degradation of adjacent tissue [3,4,7]. Considering these factors, the performance and durability of resin matrices needs to be improved.

One solution to help with the low physical and mechanical properties is to use an efficient photoinitiation system in the resin matrix. During initiation, camphorquinone (CQ) undergoes hydrogen absorbance which is a type of photoinitiation mechanism where the CQ photosensitizer absorbs light to form a photoexcitation complex with a tertiary amine. As a result, amine-derived free radicals are subsequently generated [8]. Commercial dental resin products are currently based on this system, and a light-curing unit that emits a blue color is designed to react with the yellow sensitizer, CQ. Although these photoinitiators comprise only a small amount of the resin matrix, they can affect the degree of conversion, polymerization shrinkage, color stability, mechanical properties, and clinical success [9,10]. As such, many researchers have tried to diversify the composition or content ratio of photoinitiators and the photoinitiation system itself for light curing [2,10,11,12,13,14].

Accordingly, the effectiveness of a two-component electron transfer initiator system, which can be enhanced by the addition of a third component, such as an iodonium salt, has been investigated in a number of recent studies. A three-component photoinitiator system containing methylene blue as a light absorbing molecule, *N*-methyl-diethanolamine (MDEA) as an electron donor, and diphenyliodonium chloride (DPI) as the third component, has been studied previously [15]. The neutral methylene blue radical undergoes an electron transfer to form the iodonium salt in a secondary reaction step, and DPI can be an effective electron acceptor in this system. Once the iodonium salt accepts an electron, it undergoes rapid unimolecular fragmentation which prevents electron re-transfer. In a secondary reaction step, DPI consumes an inactive methylene blue radical to produce an active phenyl radical, and simultaneously regenerate the original methylene blue dye, which leads to enhanced effectiveness of the three-component initiator system for the production of active free-radical centers. The initiator systems using iodonium salts as additives have been reported to consistently induce higher intensities than the two-component initiator system [16] and increase the efficacy of dentin bonding performance [17,18]. In addition, resistance to water solubility [18], compatibility with epoxy-based resin composites [19], and a significant increase in the polymerization rate [10] which enables fast curing can be obtained by the reaction with iodonium salts.

To date, many researchers have investigated the effectiveness of iodonium salts and ternary photoinitiator systems, but no studies have investigated bonding resins with different photoinitiation systems used in dental adhesives. Therefore, the present study focuses on modifying photoinitiator systems with iodonium salts to improve the physical and mechanical properties of the dental adhesives. The degree of conversion was measured by real time Fourier transform infrared spectroscopy (FT-IR). The flexural strength and modulus were evaluated by a universal testing machine. Microhardness was measured with a dynamic microhardness tester. The ultimate tensile strength was tested with a microtensile tester. Each measurement was performed among experimental groups with different photoinitiators. The contribution of each component to the degree of conversion within a group composed of the most effective photoinitiators was analyzed by contour plots.

## 2. Materials and Methods

### 2.1. Resin Components

Neat resin consisted of 60% bis[4-2(2-hydroxy-3-methacryloxypropoxy)phenyl]propane (Bis-GMA) and 40% hydroxyethyl methacrylate (HEMA) by weight. All compounds were purchased from Esstech (Essington, PA, USA). Five kinds of adhesive resins were fabricated by mixing the neat resin with different photoinitiators. The commercial raw materials and mixing ratios are provided in Table 1.

Groups 1 and 2 were binary systems with different tertiary amine groups. Groups 4 and 5 were ternary systems using diphenyliodonium hexafluorophosphate (DPIHFP) as the iodonium salt. Group 3 used DPIHFP and lacked a tertiary amine. The resin matrix was stirred with a magnetic stir bar for 1 h in a glass bottle in the dark. Each group was polymerized with a light-curing unit from 3M ESPE (St Paul, MN, USA, 540 mW/cm^2^) according to the instructions.

### 2.2. Degree of Conversion (DC)

Real-time polymerization was performed in an FT-IR spectrometer (Nicolet 6700, Thermo scientific, West Palm Beach, FL, USA). Spectra were obtained over the 4000–600 cm^−1^ region and acquired with a resolution of 4 cm^−1^ for a total of 32 scans per spectrum. An adhesive resin in each group was placed separately on the attenuated total reflectance (ATR) module. The FT-IR spectrum was recorded after exposure to light for 20 s (total recording time: 4 min) utilizing a dental light-curing unit. For each spectrum, the height of the aliphatic C=C peak absorption at 1636 cm^−1^ and the aromatic C–C peak absorption at 1581 cm^−1^ was determined using a baseline method. The aromatic C–C vibration was used as an internal standard. The ratio of absorbance intensities was calculated for each group and compared. The DC at each irradiation time was calculated by using the following equation:

$$\text{Degree of conversion}\left(\%\right)=100\;\times \;\frac{C\;=\;\text{C}\;cured\;/\;\text{Aromatic}\;cured}{C\;=\;C\;uncured\;/\;\text{Aromatic}\;uncured}$$

All tests were performed independently three times.

### 2.3. Three Point Bending Test

Specimens [(25 ± 2) × (2 ± 0.1) × (2 ± 0.1) mm^3^] were made from each material according to ISO 4049 and cured in five separate 20 s steps for each side (200 s in total). Tests were performed after 24 h of water storage at 37 °C using a universal testing machine (QTest, Instron, Miami, FL, USA, crosshead speed of 0.75 mm/min) according to ANSI/ADA specification No. 27-1993. Flexural strength was calculated using *σ* = (3*FL*)/(2*bh*^2^) and flexural modulus by *E* = (*L*^3^/4*bh*^3^) × (*F*/*Y*) (both expressed in MPa), where *F* is the maximum strength, *L* the distance between rests (20 mm), *b* the width of the specimen, *h* the height of the specimen, and *F*/*Y* the slope of the linear part of the stress-strain curve. Ten specimens were made and tested in each group.

### 2.4. Microhardness

The specimens in each group were fabricated by placing the adhesive resin in a stainless steel mold (15 mm in diameter and 1 mm thick) which was inserted between two sheets of clear matrix and photoactivated for eight separate 20 s steps using a light-curing unit. Surface hardness was tested using a dynamic ultra-microhardness tester (DUH-W201S, Shimadzu, Kyoto, Japan) followed by ASTM E384. Electromagnetic force was used to press an indenter (standard type: 115° triangular pyramid indenter) against a specimen. The pressing force was increased at a constant rate from 0 to the preset test force (100 mN). The holding time of the maximum load was 2 s and the loading speed was 13.2 mN/s. The indentation depth was measured automatically as the indenter pressed against the specimen, allowing a dynamic measurement of the changes occurring in the specimen’s resistance to deformation during the indentation process. Microhardness was calculated as 3.8584 × *F*/*h*^2^, where *F* is the maximum load (mN) and *h* is the maximum indentation depth (μm). Three specimens were made for each group and each test repeated three times for each specimen (total: 9 times).

### 2.5. Ultimate Tensile Strength

A microtensile strength tester (Bisco, Inc., Schaumburg, IL, USA) tested I-shaped specimens (1 mm width and depth in the center) by ASTM D1708-13. A cyanoacrylate material was used to bond the ends of each specimen to the two free-sliding parts of a specially designed holding device. The jig was able to transmit purely tensile forces to the specimen without any torqueing or bending component. The tensile load was applied at a crosshead speed of 0.5 mm/min until the specimen fractured. The weight (in kilograms) of the loaded force was recorded and the ultimate tensile strength (in MPa) calculated based on the measured width and depth (in mm) of the specimens. After the tests, the specimens were inspected by a microscope (100×) to exclude those with internal voids. All tests were performed 10 times in every group.

### 2.6. Adhesive Resins with Selected Photoinitiators

CQ, EDMAB, and DPIHFP (i.e., Group 5) were selected as the most effective photoinitiators for the adhesives after performing the DC, flexural strength, flexural modulus, microhardness, and ultimate tensile strength tests in five groups. To investigate the contribution of each material to the DC, the molar ratios of the three components were changed as shown in Table 2. After curing for 20 s using FT-IR, the conversion rate to polymer was calculated as described above. Three independent experiments were performed.

### 2.7. Statistical Analysis

One-way analysis of variance was used (α = 5%) among groups to determine significant differences. Pair-wise multiple comparisons were carried out using the Tukey test when the one-way analysis of variance test detected significant differences.

## 3. Results

### 3.1. Degree of Conversion

**Figure 1:**
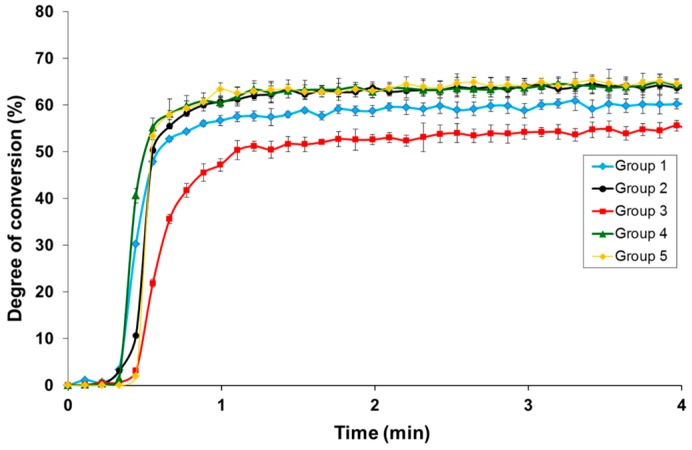
Degree of conversion in the five different groups. The maximum for Groups 2, 4, and 5 were 64.49%, 65.95%, and 66.87%, respectively. Group 3, lacking tertiary amine, had the lowest value among them.

Figure 1 shows the DC for the five kinds of adhesive resins. Groups 2, 4, and 5 had nearly the same conversion rates with maximum average values in real-time FT-IR of 64.55%, 64.56%, and 65.32%, respectively. Group 3 lacked a tertiary amine and had the lowest DC (55.58%). Group 1 had an intermediate value.

### 3.2. Three Point Bending Test

**Figure 2:**
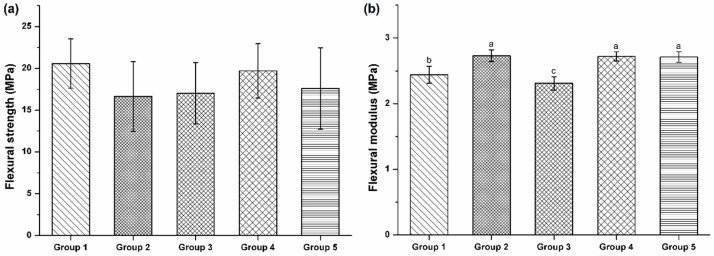
Flexural strength and modulus of the five experimental groups. (**a**) Flexural strength was not significantly different (*p* > 0.05, Tukey HSD test); (**b**) The elastic modulus of Group 3 was significantly lower than that of the other groups, whereas Groups 2, 4, and 5 were similar (*p* > 0.05, Tukey HSD test).

The flexural strengths of the five adhesive resin groups were not significantly different (*p* > 0.05, Figure 2a). However, the flexural modulus was similar and highest among Groups 2, 4, and 5, followed by Group 1 and Group 3 (*p* < 0.05, Figure 2b).

### 3.3. Microhardness

**Figure 3:**
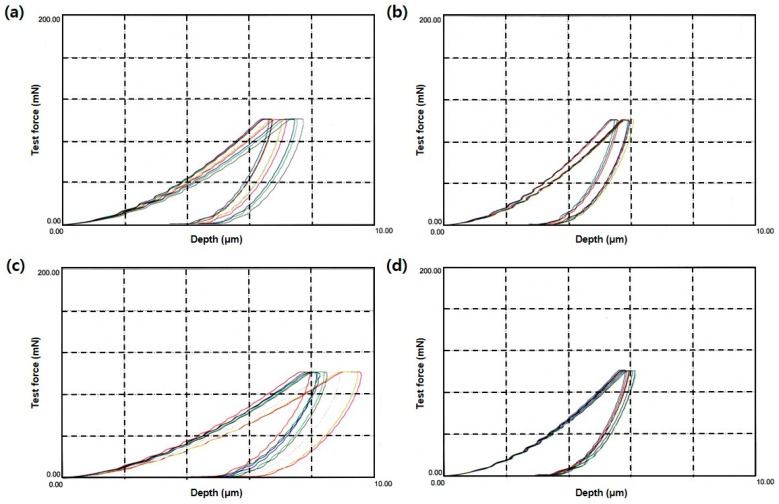

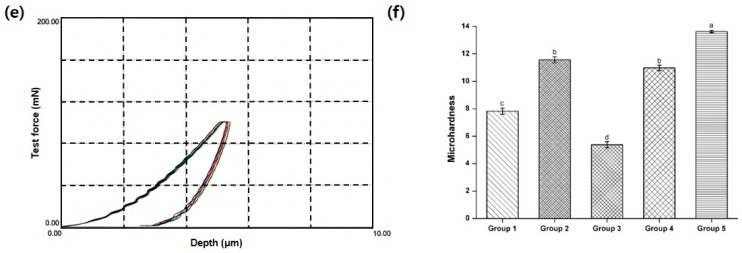
Microhardness of the five experimental groups. (**a**–**e**) Dynamic microhardness of Group 1–5, respectively; (**f**) Comparison of microhardness when maximum force is loaded. Group 3 had the lowest microhardness and Group 5 the highest (*p* < 0.05). Groups 2 and 4 were not significant different (*p* > 0.05, Tukey HSD test). Different colors in a–e means every test results.

The dynamic microhardness of the adhesive resins based on the differences in maximum indentation depth and elastic/plastic deformation is shown in Figure 3a–e. A summary of the microhardness values is displayed in Figure 3f. Group 5 had the highest microhardness (*p* < 0.05) and Group 3 had the lowest microhardness (*p* < 0.05). Groups 2 and 4 had similar values (*p* > 0.05). The differences in microhardness measurements were more significant among the groups than differences from other measurement methods.

### 3.4. Ultimate Tensile Strength

**Figure 4:**
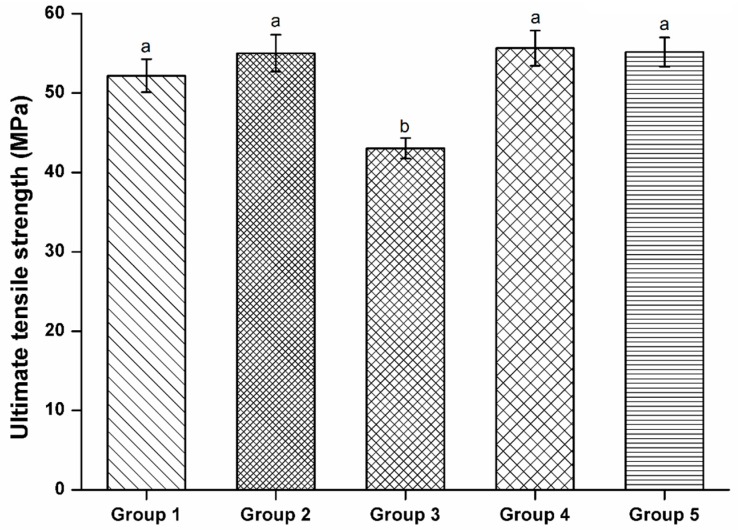
Ultimate tensile strength of the five experimental groups. Groups 1, 2, 4, and 5 were not significantly different from one another (*p* > 0.05, Tukey HSD test). Group 3 had significantly lower ultimate tensile strength compared to any of the other groups (*p* < 0.05).

None of the specimens had defects on the fractured surfaces after the tests as evaluated by a microscope. The microtensile strength of Groups 2, 4, and 5 were not significantly different (*p* > 0.05, Figure 4). Group 3 had the lowest strength (*p* < 0.05).

### 3.5. Contribution of Each Photoinitiator to the DC in the Selected Group

**Figure 5:**
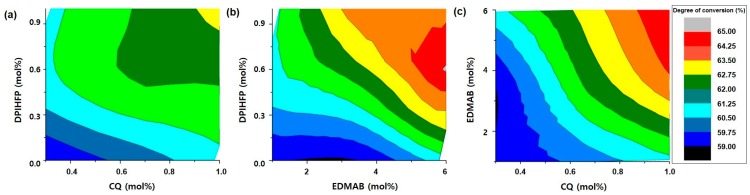
Contour plots for 27 kinds of resin combinations with different molar ratios of CQ, EDMAB, and DPIHFP. The area with the same degree of conversion was painted the same color when EDMAB was set to 1 mol % (**a**), CQ to 0.3 mol % (**b**), and DPIHFP to 0 mol % (**c**). (**a**) had a relatively wider contour plot, whereas (**b**) and (**c**) exhibited more narrow areas.

The contour plots in Figure 5 describe the relationship between two chosen components. The areas of the same color indicate identical conversion rates when the remaining photoinitiator is set to the lowest molar ratio level: EDMAB = 1 mol % (Figure 5a), CQ = 0.3 mol % (Figure 5b), and DPIHFP = 0 mol % (Figure 5c). As shown in Figure 5, CQ was the most important factor for DC followed by DPIHFP and EDMAB. When EDMAB was set to 1 mol % (Figure 5a), the DC did not change significantly, showing a wide width with the same colors. When CQ was set to 0.3 mol % (Figure 5b), the color width was much narrower than the others. Without DPIHFP, the DC was 59%–63%. When DPIHFP was present in the maximum amount, the DC increased to 67%.

## 4. Discussion

Dental adhesives are often used before applying polymer-based filling resins or other kinds of esthetic restorations in order to completely seal and protect the tooth from chemical/physical stimulation and marginal leakage. Adhesives are composed of methacrylate-based monomers that undergo free-radical polymerization and provide adhesion between a restoration and the natural tooth. Thus far, an evaluation of the physical and mechanical properties of the adhesives has been considered prior to the adaptation. One solution could be a change in the photoinitiators used in dentin adhesives, which in turn can affect the physical and mechanical aspects in the improvement of clinical performance [11,13,20,21].

Usually, a binary photoactivator system comprising CQ and an amine are used. Diaryliodonium salts with complex metal halides as weakly nucleophilic counter ions are efficient photoinitiators for UV-irradiated monomer systems which absorb light below 300 nm and are extensively used in paints and coatings [22]. However, curing based on visible light is preferred because commercial light-curing units used in dentistry emit 400–500 nm light. CQ can compensate for this drawback by changing the highest absorbance peak of resin composites to the visible light region [23]. Thus, the three component system is a practical and realistic method that can be easily adapted in dentistry. In this study, photoinitiators with different polymerization kinetics were investigated through various evaluation methods. The relationship among the most effective photoinitiators are expressed as contour plots.

As a result, Group 3 (1.0 mol % CQ and DPIHFP) was significantly inferior in all tests. The sensitizer lacks a tertiary amine group and cannot perform electron transfer or polymerize effectively. In the appropriate excited state, the diketone combines with the reducing agent to form an excited state complex (exiplex), which then breaks down to form reactive free radicals [24]. Group 3 had insufficient radical formation, resulting in the poorest physical and mechanical properties. Accordingly, amine products such as EDMAB or DMAEMA are most effective for proper polymerization.

EDMAB is a more stable product compared with DMAEMA because it is in a powder rather than a volatile liquid form. Previously, EDMAB was reported to have a higher double bond conversion rate than DMAEMA [25]. Group 2 (1.0 mol % CQ and EDMAB) had superior DC, flexural modulus, and microhardness values compared to those of Group 1 which contained DMAEMA. Group 4 contained CQ, DMAEMA, and DPIHFP and had similar DC, flexural strength, flexural modulus, and ultimate tensile strength, but had a larger microhardness value when compared with Group 5 (CQ, EDMAB, and DPIHFP). In conclusion, a ternary system is less sensitive to the selection of DMAEMA or EDMAB as an electron donor compared with a binary system. However, a photoinitiator system with EDMAB as an electron donor is more likely to possess superior physical and mechanical properties. Therefore, a proper tertiary amine (i.e., EDMAB) has to be selected prior to the comparison of a two- or three-component system.

In contrast to flexural strength and ultimate tensile strength, the microhardness tests had clear differences among the five groups. These results were in accord with previous studies [26,27]. It was also proven that dynamic indentation as controlled by a computer program resulted in obvious differences and relatively little standard deviation among groups [28]. Thus, this protocol should be additionally performed with other testing methods like the three point bending test and DC to detect definitive physical and mechanical differences among resin combinations.

A three-component system containing iodonium salts as the third factor could result in a higher DC and enhanced mechanical properties compared with a two-component system. The results presented here were in accordance with previous observations [23]. In general, Group 5 had an excellent DC, flexural modulus, microhardness, and ultimate tensile strength. From these results, CQ, EDMAB, and DPIHFP were chosen as effective photoinitiators, and further studies with different molar ratios were performed. Each was a necessary component for effective polymerization, but CQ was determined to be the most influential factor by effectively changing the DC in the contour plots.

The silanized fillers (about 10 wt %) could be mixed with the resin matrix for the resin adhesives. Generally, it has been known that enough filler can reduce polymerization shrinkage, improve resistance to external forces, and increase the durability of filling resins (packable or flowable resins). As a result, it is said that fillers improve the biocompatibility of resins by reducing the release of monomers [29,30,31,32,33]. Kim et al., however, did not find significant differences in the DC of adhesives with 0.5%–3% filler [34]. As such, it was assumed that addition of filler to the adhesive resins would not affect the DC so they were not instituted in these studies. Otherwise, Halvorson et al. observed that the DC of composites vary inversely with the percentage of filler in the material [35]. The researchers determined that the fillers might scatter light which can hinder light penetration. This is especially problematic when particle size approaches the output wavelength of the light-curing unit [36,37]. Their results were opposed to our initial hypothesis. Future studies should address these effects with micro- or nanofillers combined with different photoinitiator systems directly.

The durability of a photoinitiator system with DPIHFP is an important factor for evaluating the clinical usages of the photoinitiation system. In the previous study, a ternary photoinitiator system with iodonium salts maintained increased bond strength with dentin in a model of self-etching adhesive system after 1 year of aging [17]. Improvement was also observed in the polymerization kinetics of a model of dental adhesive resin using a ternary photoinitiator system which made the material less sensitive to the residual presence of a solvent before photoactivation [38]. The yellow effect of CQ could be reduced by the white color of the powdered iodonium salts additionally. In the further studies, the dental adhesives with alternative photoinitiators in this study need to be evaluated with respect to color stability, durability, degradation rate after polymerization, and the deformation examination after hybrid formation with different solvents in an animal experiment.

Despite these limitations, this study showed the effectiveness of the EDMAB as a tertiary amine and ternary photoinitiator system in dental adhesives. Additionally, dynamic microhardness tests were capable of identifying clear differences in properties among test groups. Additionally, it was proven that the molar ratio of CQ was the most influential on DC rate.

## 5. Conclusions

This study found that dental adhesives with EDMAB had better mechanical and physical properties as compared with adhesives containing DMAEMA. Also, iodonium salts, DPIHFP, did not affect the mechanical and physical properties without amine initiators. Dynamic microhardness tests were a more effective test method than the flexural strength and ultimate tensile strength tests for investigating the differences among photoinitiator systems. It was demonstrated that the molar ratio of CQ was the most influential factor on the DC rate in the present study.

## Figures and Tables

**Table 1:** Photoinitiator systems used in each experimental group.

	CQ (mol%)	DMAEMA (mol%)	EDMAB (mol%)	DPIHFP (mol%)
Group 1	1.0	1.0	-	-
Group 2	1.0	-	1.0	-
Group 3	1.0	-	-	1.0
Group 4	1.0	1.0	-	1.0
Group 5	1.0	-	1.0	1.0

Neat resin; 60% Bis-GMA + 40% HEMA (by weight %); Bis-GMA (2,2-bis[4-2(2-hydroxy-3-methacryloxypropoxy)phenyl] propane, Cook, San Clemente, CA, USA, Lot no. 10-12740, *M*_W_ 512.59 g/mol); TEGDMA (triethyleneglycol dimethacrylate, Sartomer, Exton, PA, USA, Lot no. 11-3866, *M*_W_ 286 g/mol); CQ (Camphorquinone, Sartomer, Lot no. 900009767, *M*_W_ 166.12 g/mol); DMAEMA (2-(dimethylamino)ethyl methacrylate, Sigma-aldrich, St. Louis, MO, USA, 46396APV, *M*_W_ 157.21 g/mol); EDMAB (ethyl-4-(dimethylamino) benzoate, Sartomer, Lot no. 11-5377. *M*_W_ 193.24 g/mol); DPIHFP (diphenyliodonium hexafluorphosphate, Sartomer, Lot no. 05-7979. *M*_W_ 426.08 g/mol).

**Table 2:** Photoinitiator combinations for investigating an influential index.

	CQ (mol%)	EDMAB (mol%)	DPIHFP (mol%)
I	0.30	1.00	0.00
II	0.30	1.00	0.50
III	0.30	1.00	1.00
IV	0.30	3.50	0.00
V	0.30	3.50	0.50
VI	0.30	3.50	1.00
VII	0.30	6.00	0.00
VIII	0.30	6.00	0.50
IX	0.30	6.00	1.00
X	0.65	1.00	0.00
XI	0.65	1.00	0.50
XII	0.65	1.00	1.00
XIII	0.65	3.50	0.00
XIV	0.65	3.50	0.50
XV	0.65	3.50	1.00
XVI	0.65	6.00	0.00
XVII	0.65	6.00	0.50
XVIII	0.65	6.00	1.00
XIX	1.00	1.00	0.00
XX	1.00	1.00	0.50
XXI	1.00	1.00	1.00
XXII	1.00	3.50	0.00
XXIII	1.00	3.50	0.50
XXIV	1.00	3.50	1.00
XXV	1.00	6.00	0.00
XXVI	1.00	6.00	0.50
XXVII	1.00	6.00	1.00
